# Supplementary material for: Cotranslational N-degron masking by acetylation promotes proteome stability in plants
Source: Nat Commun. 2022 Feb 10;13:810. doi: 10.1038/s41467-022-28414-5 (PMC8831508; doi:10.1038/s41467-022-28414-5)
Supplement: Supplementary file 1 — Supplementary Information [file 41467_2022_28414_MOESM1_ESM.pdf]

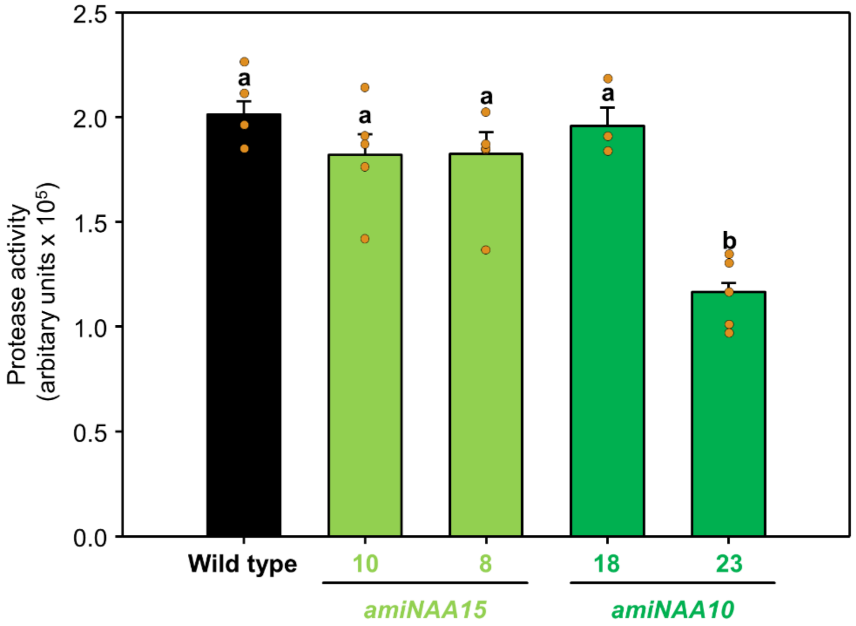

Supplementary Fig. 1. Protease activity in leaves of wild type and NatA depleted plants. Quantification of metallo-, serine-, acid-, and sulfhydryl-type protease activity with the EnzChek™ protease assay kit in leaves of five-week-old wild type and two transgenic lines depleted for the ribosome anchoring (*amiNAA15*) or the catalytically active subunit of the NatA complex (*amiNAA10*). Different letters indicate individual groups identified by pairwise multiple comparisons with a Holm-Sidak, one-way ANOVA ($p < 0.05$, n=4 biologically independent samples, except for *amiNaa10* #18 n=3). Data are shown as mean ± standard error.

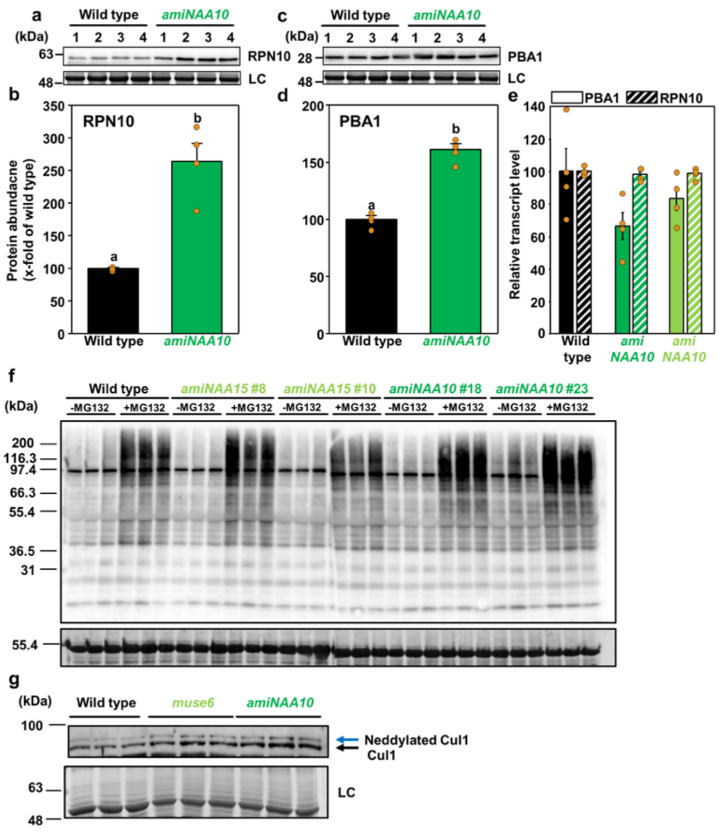

Supplementary Fig. 2. Immunological detection of proteasome subunits, poly-ubiquitinylated proteins and Cullin 1 in leaves of wild type and NatA depleted plants

a, **b** Immunological detection of the 26S proteasome regulatory subunit (RPN10, At4g38630) and the core proteasome beta subunit 1 (PBA1, At4g31300) in leaves of wild type and transgenic plants depleted of the catalytic subunit of NatA (*amiNAA10*) with specific antibodies. Different letters indicate individual groups identified by pairwise multiple comparisons with a Holm-Sidak, one-way ANOVA (n=4 biologically independent samples). Data are shown as mean ± standard error.

c ,**d** Quantification of RPN10 (c) or PBA1 (d) in leaves after normalization to the loading control (LC). The abundance of RPN10 or PBA1 in the wild type was set to 1. Data represent mean ± standard error. Different letters indicate individual groups identified by pairwise multiple comparisons with a Holm-Sidak, one-way ANOVA ($p < 0.05$, n=4 biologically independent samples). Data are shown as mean ± standard error. **e**, Steady-state transcript levels of RPN10 and PBA1 in leaves of wild type and transgenic plants depleted of the catalytic subunit of NatA (*amiNAA10*) as determined by qRT-PCR (n=4 biologically independent samples). Data are shown as mean ± standard error. **f**, Immunological detection of poly-ubiquitinylated soluble protein in leaves of 5-week old soil grown wild type and transgenic plants depleted of the catalytic subunit NAA10 (*amiNAA10*) or the ribosome anchoring subunit NAA15 (*amiNAA15*) of NatA. Plants were treated with (+MG132) or without (-MG132) proteasome inhibitor for 4 hours before protein extraction for detection of poly-ubiquitinated proteins with the ubiquitin-specific antiserum (UBQ11, Agrisera). Quantification of the signal after normalization to the loading control (LC) is shown in Figure 1c. (n=3 biologically independent samples) **g**, Immunological detection of cullin1 (black arrow) and neddylated cullin1 (blue arrow) in leaves of 6-week old soil grown wild type and NatA depleted *muse6* and *amiNAA10* plants (upper panel). The lower panel shows the corresponding loading control (LC). Quantification of CUL1 and neddylated CUL1 signal intensities are shown in Figure 1d (n=3 biologically independent samples). Immunological detections of proteins quantified in panel b and d were independently repeated and blots are shown in the source data file.

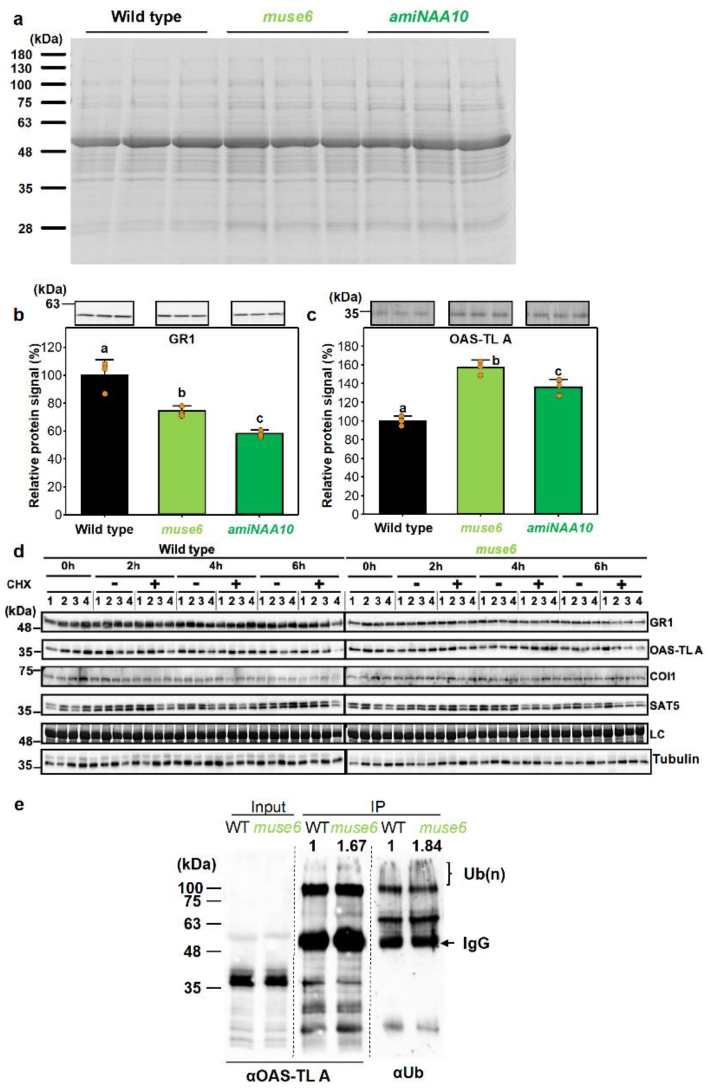

Supplementary Fig. 3. Steady-state levels and stability of selected soluble proteins extracted from leaves of wild type and NatA depleted plants

a, Soluble proteins were extracted from leaves of 6-week-old wild type and transgenic plants depleted for the catalytic (*amiNAA10*) or the ribosome anchoring subunit of NatA (*muse6*). Identical volumes of these extracts were separated by SDS-polyacrylamide gel electrophoresis and proteins were stained with Coomassie blue (n=3 biologically independent samples). **b**, **c** Verification of ^{ARK}GR1 (b) and ^{ASR}OAS-TL A (**c**) protein steady state levels in leaves of six-week-old wild type and transgenic plants depleted for the catalytic (*amiNAA10*, dark green) or the ribosome anchoring subunit of NatA (*muse6*, light green) with specific antisera. Data represent mean ± standard deviation. Different letters indicate individual groups identified by pairwise multiple comparisons with a Holm-Sidak, one-way ANOVA ($p < 0.05$, n=3 biologically independent samples) **d**, Western blots for time-resolved degradation analysis of selected NatA substrates (GR1, OAS-TL A) and proteins that are not N-terminally acetylated by NatA (COI1, SAT5) in the wild type *muse6* in presence (+) or absence (-) of the translation inhibitor cycloheximide (CHX). Quantification of signal based on the loading control (LC) is shown in Figure 2c,d. (n=4 biologically independent samples) **e**, Confirmation of enhanced poly-ubiquitination of OAS-TL A in NatA depleted plants (*muse6*) after affinity purification with a specific OAS-TL A serum. The level of poly-ubiquitinated OAS-TL A was detected in the enriched fraction (IP) with selective antibodies against OAS-TL A (αOAS-TL A) and mono/poly-ubiquitination (αUB) as described in material and methods (n=2 biologically

independent samples). Immunological detections of proteins quantified in panel e were independently repeated and blots are shown in the source data file.

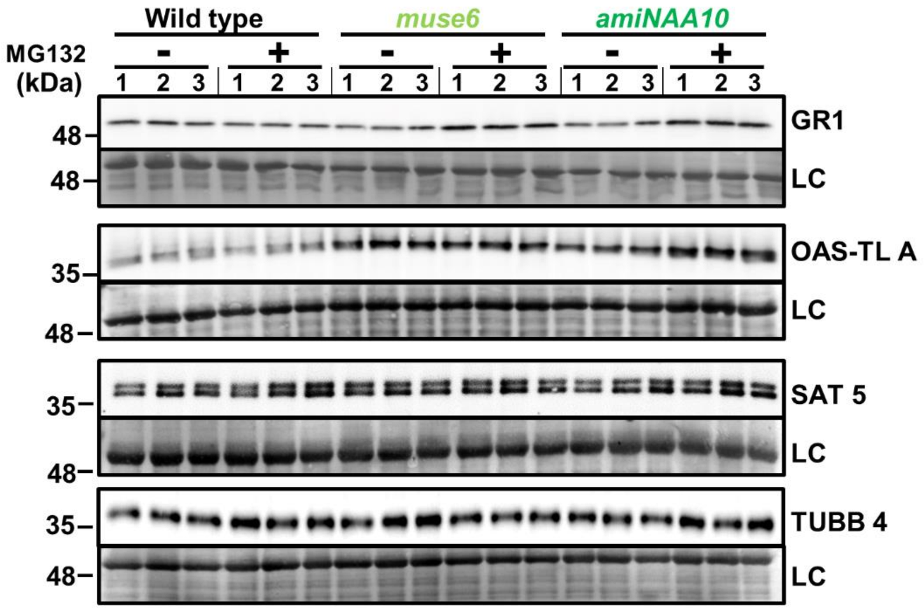

Supplementary Figure 4. Accumulation of selected proteins in leaves of wild type and NatA depleted plant after inhibition of the proteasome.

Protein steady-state levels of ^{ASR}OAS-TL A, ^{ARK}GR1, ^{MPP}SAT5 and ^{MRE}TUBB4 as determined with the specific antisera (α-UBQ11; Agrisera) in leaves of wild type and transgenic lines depleted of the ribosome-anchoring NatA subunit NAA15 (*muse6*) and the catalytically active NatA subunit NAA10 (*amiNAA10*) in presence (+) or absence (-) of the proteasome inhibitor MG132 (n=3 biologically independent samples). Quantification of signal based on the loading control (LC) is shown in Figure 2e.

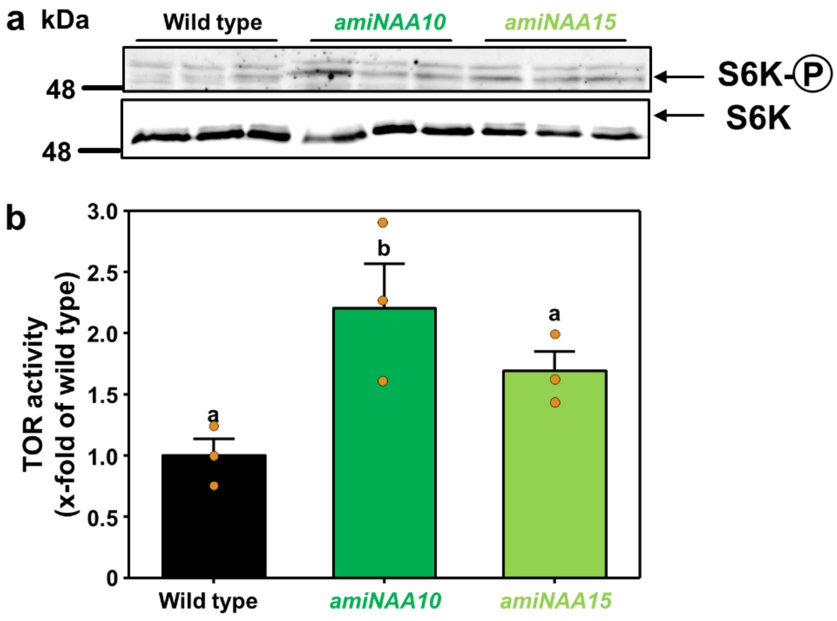

Supplementary Fig. 5. Target of Rapamycin (TOR) activity in leaves of wild type and NatA depleted plants

a, Immunological detection of the ribosomal S6 kinase (S6K) and the phosphorylation of T^{449} of S6K by TOR using specific antibodies in leaves of 5-week-old wild type and transgenic plants depleted of the catalytic (*amiNAA10*) or the ribosome anchoring subunit of NatA (*amiNAA15*). **b**, TOR activity was calculated by determining the ratio of phosphorylated S6K (S6K-P) to S6K and set to 1 in the wild type. Data represent mean ± standard error. Different letters indicate individual groups identified by pairwise multiple comparisons with a Holm-Sidak, one-way ANOVA ($p < 0.05$, n=3 biologically independent samples)

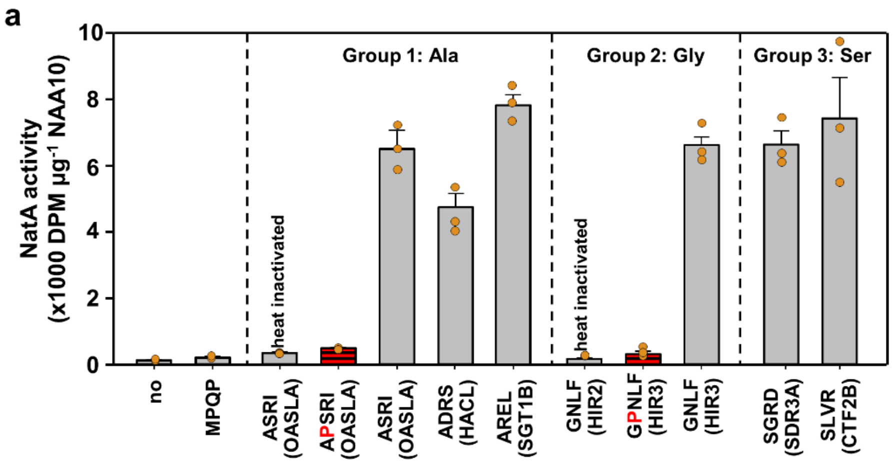

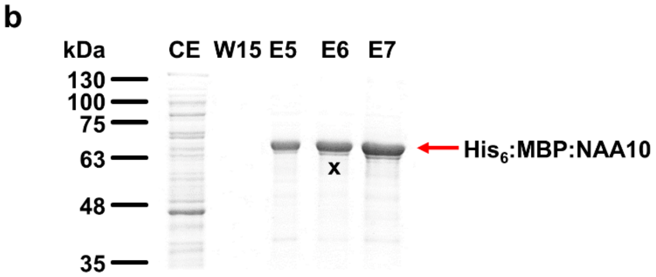

Supplementary Fig. 6. Confirmation of candidate NatA substrates and proline-induced inhibition of N-terminal acetylation of NatA substrate candidates by in vitro NatA activity tests

a, Purified recombinant full-length His$_6$MBP-NAA10 was applied to test for *in vitro* N-terminal acetylation activity on peptides representing the N-termini of selected NatA substrate candidates shown in brackets: ASRI (OASTL A), GNLF (HIR3), ADRS (HACL), AREL (SGT1B), SGRD (SDR3A) and SLVR (CTF2B) peptides. The activity of the His6-MBP-NAA10 protein on an iMet-starting peptide, MPQP, and in the absence of peptide (no) were determined as negative controls. As additional specificity controls, the His6-MBP-NAA10 protein was heat-inactivated prior incubation with the ASRIA or the GNLF peptide (heat inactivated). The inhibition of substrate recognition by the catalytic subunit of NatA due to engineering of a proline at position 3 in context of peptides starting with an A (APSRI) or a G (GPNLF) has been experimentally confirmed (red dashed). Proline-mediated inhibition of substrate recognition by NatA in context of S starting peptides was previously shown in Supplementary Figure 1b of Linster, Stephan [1]. Data represent mean ± standard error (n=3 independent samples). **b**, Enrichment of the His$_6$-MBP-NAA10 fusion protein used for the *in vitro* assay by immobilized metal affinity chromatography (IMAC). Proteins of the *E. coli* crude extract (CE), a representative washing fraction (W15) and representative fractions of the imidazole triggered His6-MBP-NAA10 elution (E5 – E7) were separated by SDS-PAGE and visualized by Coomassie-staining. The red arrow indicates the His$_6$-MBP-NAA10 (predicted MW: 66 kDa). 17 µg of elution fraction 6 (X) was used for the *in vitro* acetylation assay.

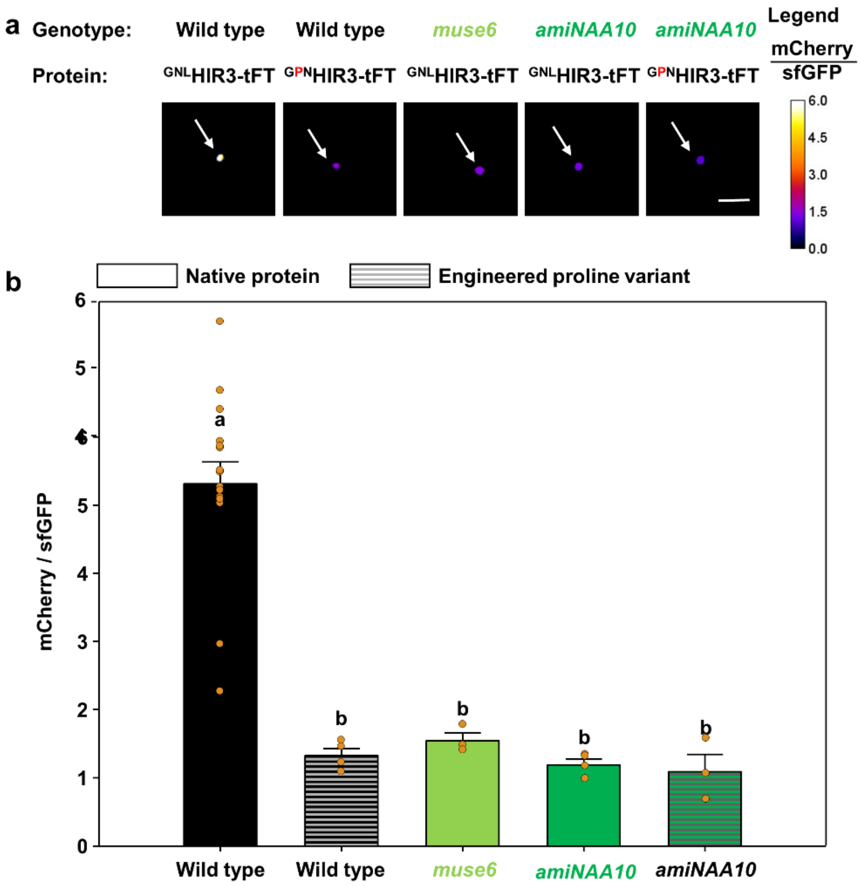

Supplementary Fig. 7a. Quantification of relative protein half-life time of the NatA substrate HIR3 in leaves of wild type and NatA depleted plants

a, False color mCherry/sfGFP signal intensity ratio of the native HIR3 protein (^{GNL}HIR3-tFT) or the non-acetylated HIR3 protein variant (^{GPN}HIR3-tFT) fused to the tandem Fluorescent Timer. The HIR3-tFT reporter is constitutively expressed in Arabidopsis pavement cells after *Agrobacterium tumefaciens*-mediated transient transformation. HIR3 is a nucleocytoplasmic protein that predominantly accumulates in the nucleus after pathogen attack, like infection with *Agrobacterium tumefaciens*. White arrows indicate the nucleus. Scale bar = 15 µM **b**, Quantification protein half-lifetime as determined by the ratio of mCherry and sfGFP fluorescence of the native ^{GNL}HIR3-tFT protein (non-shaded) or the ^{GPN}HIR3-tFT (red shaded) in leaves of wild type (black), and transgenic plants depleted for the ribosome anchoring subunit NAA15 (*muse6*, light green) or the catalytic subunit NAA10 (*amiNAA10*, dark green). Data represent mean ± standard error. Different letters indicate individual groups identified by pairwise multiple comparisons with a Holm-Sidak, one-way ANOVA ($p < 0.05$, WT ^{GNL}HIR3-tFT n=16, WT ^{GPN}HIR3-tFT n=4, *muse6* ^{GNL}HIR3-tFT n=3, *amiNaa10* ^{GNL}HIR3-tFT n=4, and *amiNaa10* ^{GPN}HIR3-tFT n=3)

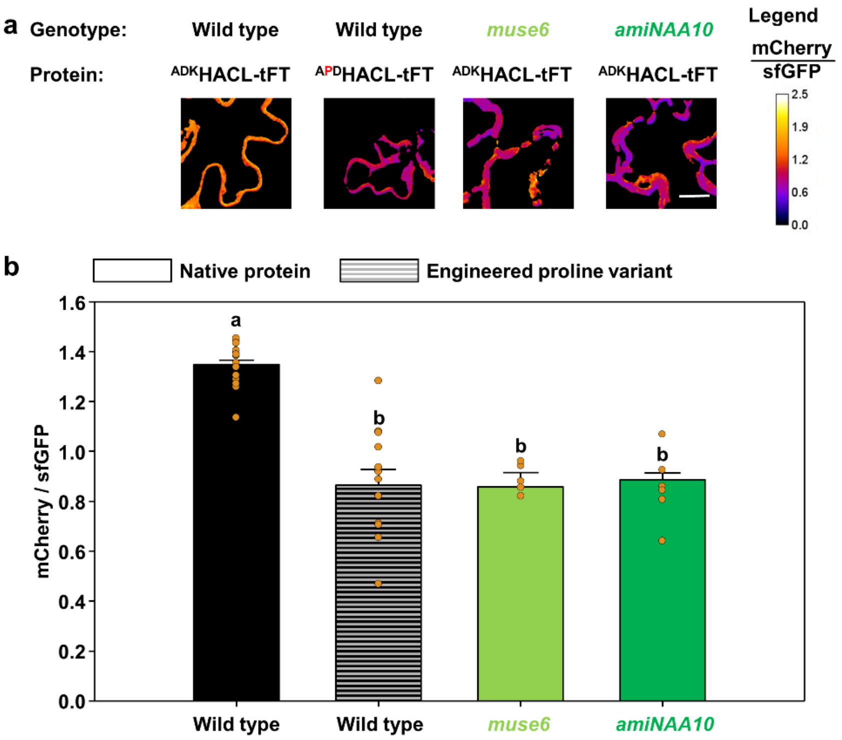

Supplementary Fig. 7b. Quantification of relative protein half-life time of the NatA substrate HACL in leaves of wild type and NatA depleted plants

a, False color mCherry/sfGFP signal intensity ratio of the native HACL protein (^{ADK}HACL-tFT) or the non-acetylated HACL protein variant (^{APD}HACL-tFT) fused to the tandem Fluorescent Timer in Arabidopsis pavement cells after *Agrobacterium tumefaciens*-mediated transient transformation. Scale bar = 15 µM. **b**, Quantification protein half-lifetime as determined by the ratio of mCherry and sfGFP fluorescence of the native ^{ADK}HACL-tFT protein (not shaded) or the ^{APD}HACL-tFT (red shaded) in leaves of wild type (black), and transgenic plants depleted for the ribosome anchoring subunit NAA15 (*muse6*, light green) or the catalytic subunit NAA10 (*amiNAA10*, dark green). Data represent mean ± standard error. Different letters indicate individual groups identified by pairwise multiple comparisons with a Holm-Sidak, one-way ANOVA ($p < 0.05$, WT ^{ADK}HACL-tFT n=19, WT ^{APD}HACL-tFT n=13, *muse6* ^{ADK}HACL-tFT n=5, and *amiNaa10* ^{ADK}HACL-tFT n=6).

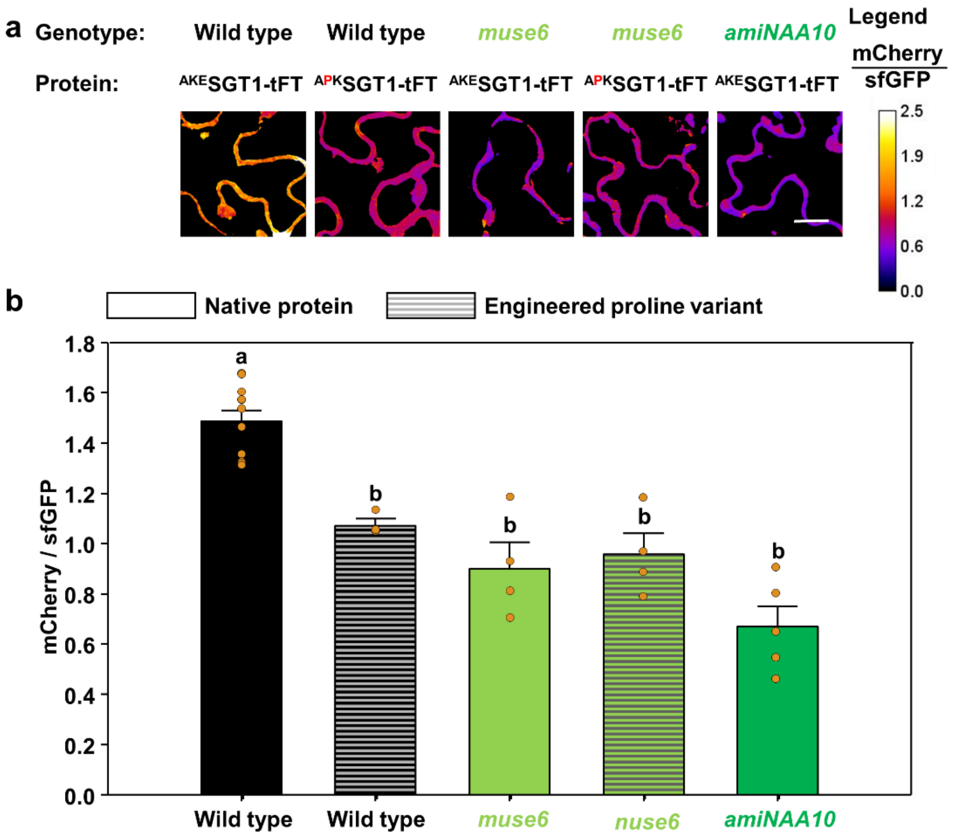

Supplementary Fig. 7c. Quantification of relative protein half-life time of the NatA substrate SGT1 in leaves of wild type and NatA depleted plants

a, False color mCherry/sfGFP signal intensity ratio of the native SGT1 protein (^{AKE}SGT1-tFT) or the non-acetylated SGT1 protein variant (^{APK}SGT1-tFT) fused to the tandem Fluorescent Timer in Arabidopsis pavement cells after *Agrobacterium tumefaciens*-mediated transient transformation. Scale bar = 15 µM. **b**, Quantification protein half-lifetime as determined by the ratio of mCherry and sfGFP fluorescence of the native ^{AKE}SGT1-tFT protein (not shaded) or the ^{APK}SGT1-tFT (red shaded) in leaves of wild type (black), and transgenic plants depleted for the ribosome anchoring subunit NAA15 (*muse6*, light green) or the catalytic subunit NAA10 (*amiNAA10*, dark green). Data represent mean ± standard error. Different letters indicate individual groups identified by pairwise multiple comparisons with a Holm-Sidak, one-way ANOVA ($p < 0.05$, WT ^{AKE}SGT1-tFT n=11, WT ^{APK}SGT1-tFT n=3, *muse6* ^{AKE}SGT1-tFT n=4, *muse6* ^{APK}SGT1-tFT n=5, and *amiNaa10* ^{AKE}SGT1-tFT n=6).

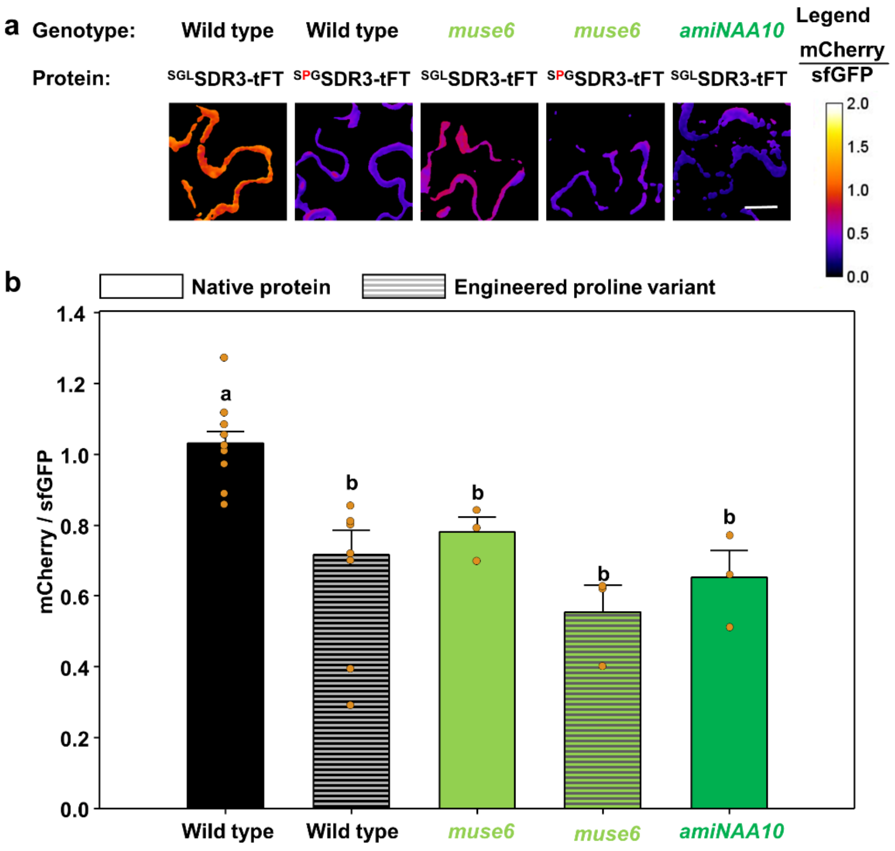

Supplementary Fig. 7d. Quantification of relative protein half-life time of the NatA substrate SDR3 in leaves of wild type and NatA depleted plants

a, False color mCherry/sfGFP signal intensity ratio of the native SDR3 protein (^{SGL}SDR3-tFT) or the non-acetylated SDR3 protein variant (^{SPG}SDR3-tFT) fused to the tandem Fluorescent Timer in Arabidopsis pavement cells after *Agrobacterium tumefaciens*-mediated transient transformation. Scale bar = 15 µM. **b**, Quantification protein half-lifetime as determined by the ratio of mCherry and sfGFP fluorescence of the native ^{SGL}SDR3-tFT protein (not shaded) or the ^{SPG}SDR3-tFT (red shaded) in leaves of wild type (black), and transgenic plants depleted for the ribosome anchoring subunit NAA15 (*muse6*, light green) or the catalytic subunit NAA10 (*amiNAA10*, dark green). Data represent mean ± standard error. Different letters indicate individual groups identified by pairwise multiple comparisons with a Holm-Sidak, one-way ANOVA ($p < 0.05$, WT ^{SGL}SDR3-tFT n=12, WT ^{SPG}SDR3-tFT n=7, *muse6* ^{SGL}SDR3-tFT n=3, *muse6* ^{SPG}SDR3-tFT n=3, and *amiNaa10* ^{SGL}SDR3-tFT n=3).

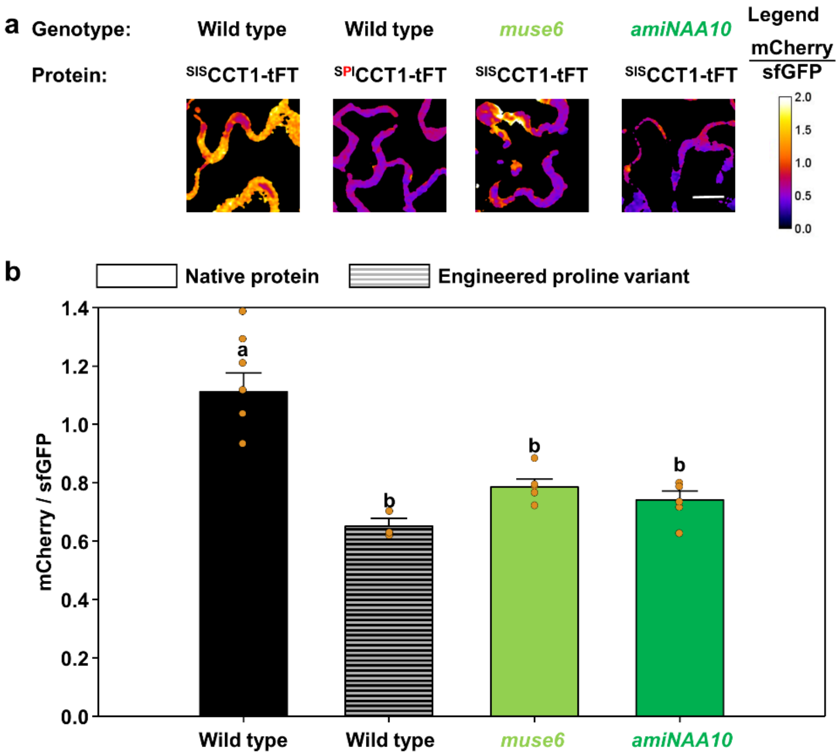

Supplementary Fig. 7e. Quantification of relative protein half-life time of the NatA substrate CCT1 in leaves of wild type and NatA depleted plants

a, False color mCherry/sfGFP signal intensity ratio of the native CCT1 protein (^{SIS}CCT1-tFT) or the non-acetylated CCT1 protein variant (^{SPS}CCT1-tFT) fused to the tandem Fluorescent Timer in Arabidopsis pavement cells after *Agrobacterium tumefaciens*-mediated transient transformation. Scale bar = 15 µM. **b**, Quantification protein half-lifetime as determined by the ratio of mCherry and sfGFP fluorescence of the native ^{SIS}CCT1-tFT protein (not shaded) or the ^{SPS}CCT1-tFT (red shaded) in leaves of wild type (black), and transgenic plants depleted for the ribosome anchoring subunit NAA15 (*muse6*, light green) or the catalytic subunit NAA10 (*amiNAA10*, dark green). Data represent mean ± standard error. Different letters indicate individual groups identified by pairwise multiple comparisons with a Holm-Sidak, one-way ANOVA ($p < 0.05$, WT ^{SIS}CCT1-tFT n=8, WT ^{SPS}CCT1-tFT n=3, *muse6* ^{SIS}CCT1-tFT n=5, and *amiNaa10* ^{SIS}CCT1-tFT n=5).

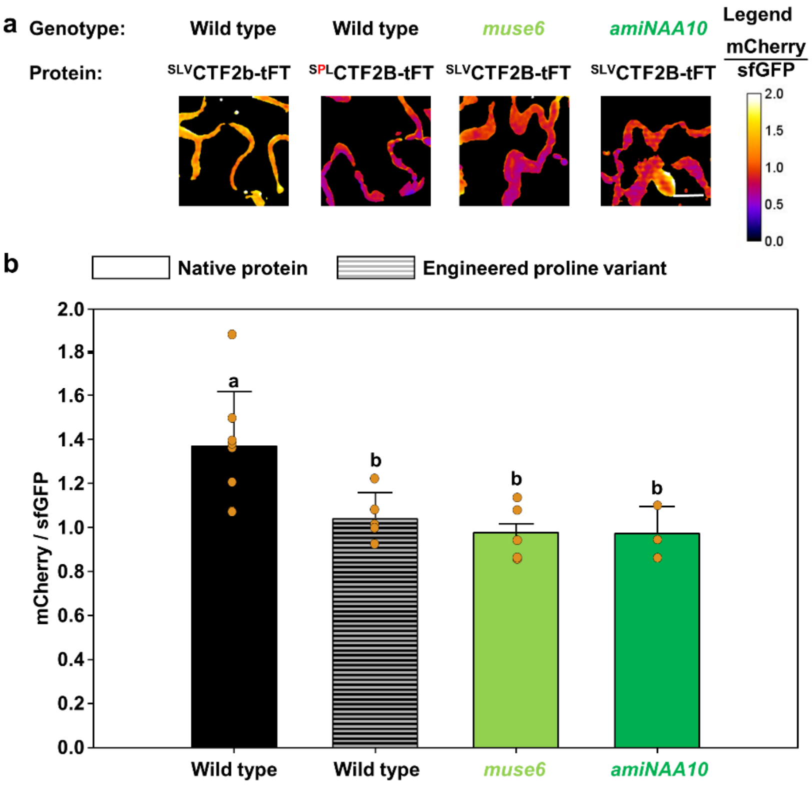

Supplementary Fig. 7f. Quantification of relative protein half-life time of the NatA substrate CTF2B in leaves of wild type and NatA depleted plants

a, False color mCherry/sfGFP signal intensity ratio of the native CTF2B protein (^{SLV}CTF2B-tFT) or the non-acetylated CTF2B protein variant (^{SPL}CTF2B-tFT) fused to the tandem Fluorescent Timer in Arabidopsis pavement cells after *Agrobacterium tumefaciens*-mediated transient transformation. Scale bar = 15 µM. **b**, Quantification protein half-lifetime as determined by the ratio of mCherry and sfGFP fluorescence of the native ^{SLV}CTF2B-tFT protein (not shaded) or the ^{SPL}CTF2B-tFT (red shaded) in leaves of wild type (black), and transgenic plants depleted for the ribosome anchoring subunit NAA15 (*muse6*, light green) or the catalytic subunit NAA10 (*amiNAA10*, dark green). Data represent mean ± standard error. Different letters indicate individual groups identified by pairwise multiple comparisons with a Holm-Sidak, one-way ANOVA ($p < 0.05$, WT ^{SLV}CTF2B-tFT n=9, WT ^{SPL}CTF2B-tFT n=5, *muse6* ^{SLV}CTF2B-tFT n=5, and *amiNaa10* ^{SLV}CTF2B-tFT n=3).

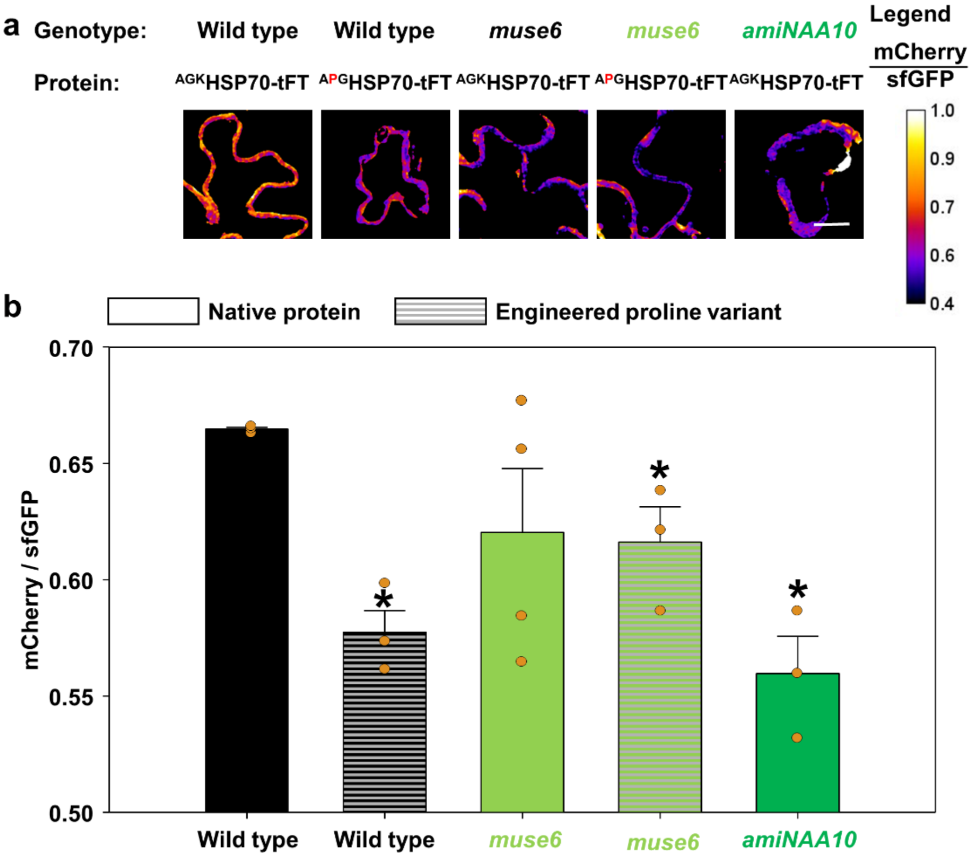

Supplementary Fig. 7g. Quantification of relative protein half-life time of the NatA substrate HSP70-2 in leaves of wild type and NatA depleted plants

a, False color mCherry/sfGFP signal intensity ratio of the native HSP70-2 protein (^{AGK}HSP70-2-tFT) or the non-acetylated HSP70-2 protein variant (^{APG}HSP70-2-tFT) fused to the tandem Fluorescent Timer in Arabidopsis pavement cells after *Agrobacterium tumefaciens*-mediated transient transformation. Scale bar = 15 µM. **b**, Quantification protein half-lifetime as determined by the ratio of mCherry and sfGFP fluorescence of the native ^{AGK}HSP70-2-tFT protein (not shaded) or the ^{APG}HSP70-2-tFT (red shaded) in leaves of wild type (black), and transgenic plants depleted for the ribosome anchoring subunit NAA15 (*muse6*, light green) or the catalytic subunit NAA10 (*amiNAA10*, dark green). Data represent mean ± standard error. Asterisx indicate significant difference towards the wild type determined by the students t-test (two-tailed $p < 0.05$, WT ^{AGK}HSP70-2-tFT n=3, WT ^{APG}HSP70-2-tFT n=3 *muse6* ^{AGK}HSP70-2-tFT n=4, *muse6* ^{APG}HSP70-2-tFT n=3, and *amiNaa10* ^{AGK}HSP70-2-tFT n=3).

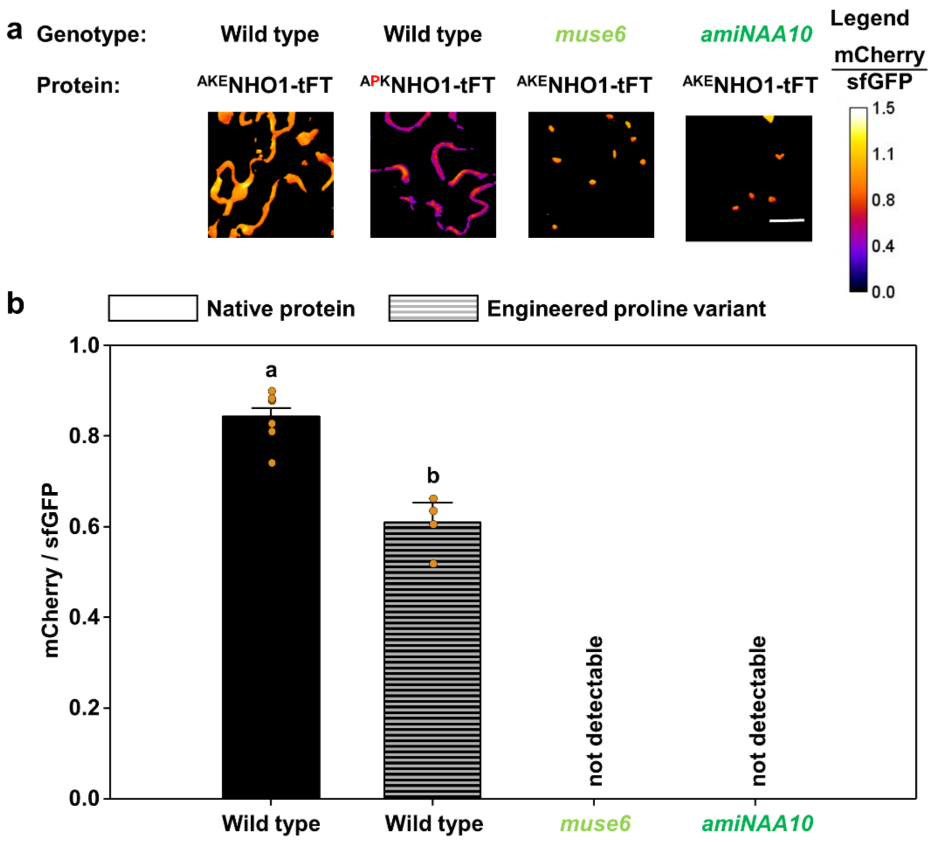

Supplementary Fig. 7h. Quantification of relative protein half-life time of the NatA substrate NHO1 in leaves of wild type and NatA depleted plants

a, False color mCherry/sfGFP signal intensity ratio of the native NHO1 protein (^{AKE}NHO1-tFT) or the non-acetylated NHO1 protein variant (^{APK}NHO1-tFT) fused to the tandem Fluorescent Timer in Arabidopsis pavement cells after *Agrobacterium tumefaciens*-mediated transient transformation. Scale bar = 15 µM. **b**, Quantification protein half-lifetime as determined by the ratio of mCherry and sfGFP fluorescence of the native ^{AKE}NHO1-tFT protein (not shaded) or the ^{APK}NHO1-tFT (red shaded) in leaves of wild type (black), and transgenic plants depleted for the ribosome anchoring subunit NAA15 (*muse6*, light green) or the catalytic subunit NAA10 (*amiNAA10*, dark green). Data represent mean ± standard error. Different letters indicate individual groups identified by pairwise multiple comparisons with a Holm-Sidak, one-way ANOVA ($p < 0.05$, WT ^{AKE}NHO1-tFT n=8, and WT ^{APK}NHO1-tFT n=3).

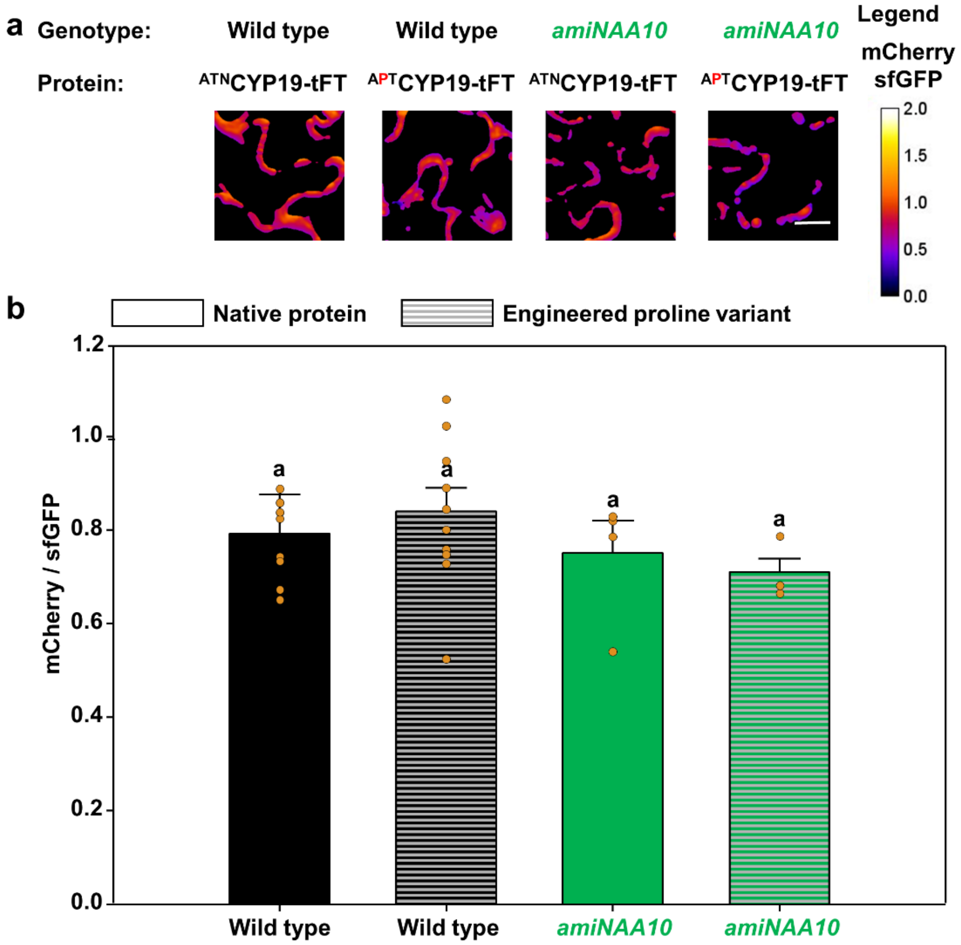

Supplementary Fig. 7i. Quantification of relative protein half-life time of the NatA substrate CYP19 in leaves of wild type and NatA depleted plants

a, False color mCherry/sfGFP signal intensity ratio of the native CYP19 protein (^{ATN}CYP19-tFT) or the non-acetylated CYP19 protein variant (^{APT}CYP19-tFT) fused to the tandem Fluorescent Timer in Arabidopsis pavement cells after *Agrobacterium tumefaciens*-mediated transient transformation. Scale bar = 15 µM. **b**, Quantification protein half-lifetime as determined by the ratio of mCherry and sfGFP fluorescence of the native ^{ATN}CYP19-tFT protein (not shaded) or the ^{APT}CYP19-tFT (red shaded) in leaves of wild type (black), and transgenic plants depleted for the ribosome anchoring subunit NAA15 (*muse6*, light green) or the catalytic subunit NAA10 (*amiNAA10*, dark green). Data represent mean ± standard error. Different letters indicate individual groups identified by pairwise multiple comparisons with a Holm-Sidak, one-way ANOVA ($p < 0.05$, WT ^{ATN}CYP19-tFT n=10, WT ^{APT}CYP19-tFT n=10, *amiNaa10* ^{ATN}CYP19-tFT n=4, and *amiNaa10* ^{APT}CYP19-tFT n=4).

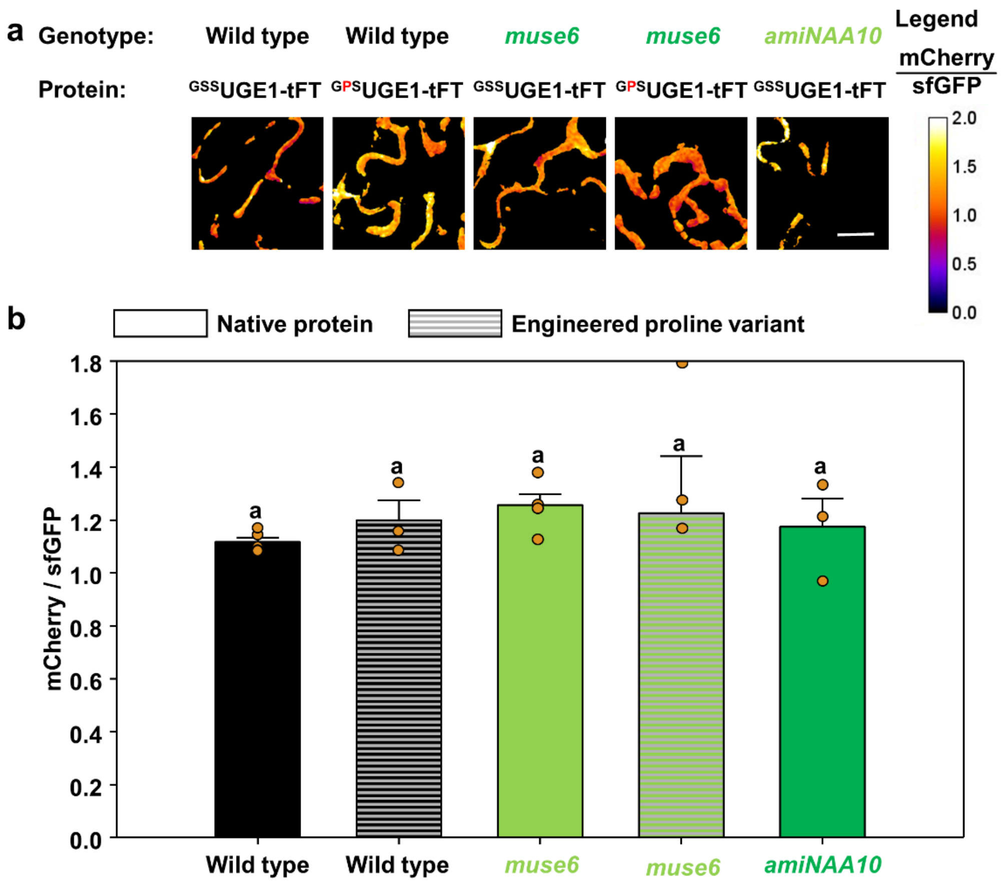

Supplementary Fig. 7j. Quantification of relative protein half-life time of the NatA substrate UGE1 in leaves of wild type and NatA depleted plants

a, False color mCherry/sfGFP signal intensity ratio of the native UGE1 protein (^{GSS}UGE1-tFT) or the non-acetylated UGE1 protein variant (^{GPS}UGE1-tFT) fused to the tandem Fluorescent Timer in Arabidopsis pavement cells after *Agrobacterium tumefaciens*-mediated transient transformation. Scale bar = 15 µM. **b**, Quantification protein half-lifetime as determined by the ratio of mCherry and sfGFP fluorescence of the native ^{GSS}UGE1-tFT protein (not shaded) or the ^{GPS}UGE1-tFT (red shaded) in leaves of wild type (black), and transgenic plants depleted for the ribosome anchoring subunit NAA15 (*muse6*, light green) or the catalytic subunit NAA10 (*amiNAA10*, dark green). Data represent mean ± standard error. Different letters indicate individual groups identified by pairwise multiple comparisons with a Holm-Sidak, one-way ANOVA ($p < 0.05$, WT ^{GSS}UGE1-tFT n=6, WT ^{GPS}UGE1-tFT n=3 *muse6* ^{GSS}UGE1-tFT n=4, *muse6* ^{GPS}UGE1-tFT n=3, and *amiNaa10* ^{GSS}UGE1-tFT n=3).

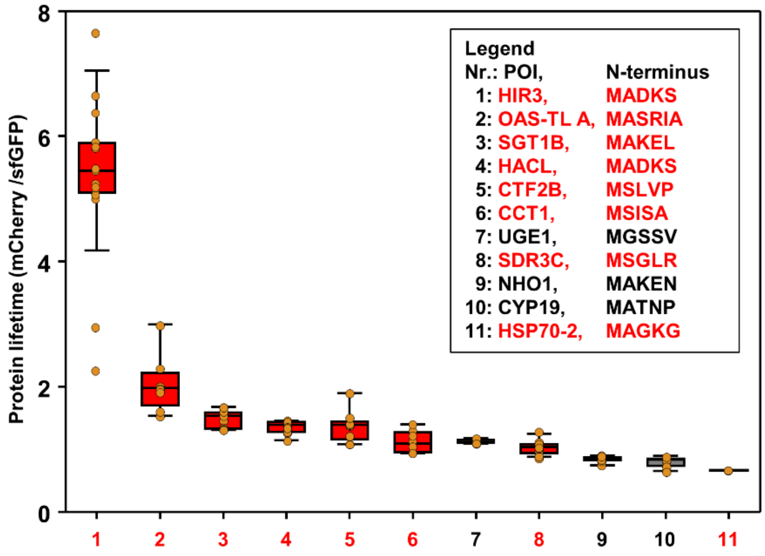

Supplementary Figure 8. Relative protein half-lifetimes of selected NatA substrates in the wild type

a, Quantification of protein half-life times with the tandem-fluorescence timer (tFT) is based on the different maturation times of the fluorescent mCherry and the super-folding green fluorescent protein (sfGFP) encoded on the same polypeptide chain fused to the protein of interest (POI-tFT: 1 - 11). The POI-tFT constructs were transiently transformed in leaves of 5-week old wild type plants. Red color indicates NatA substrates containing a nonAc-X²/N-degron. These model nonAc-X²/N-degron containing proteins are stabilized by NTA in the wild type. Data represent median values. The box boundaries represent the 25th and 75th percentile, respectively. The error bars indicate the 90th and 10th percentile. Black spheres indicate outliers (HIR3 n=16, OAS-TL A n=8, SGT1B n=11, HACL n=19, CTF2B n=9, CCT1 n=8, UGE1 n=6, SDR3 n=12, NHO1 n=8, CYP19 n=10, and HSP10-2 n=3).

Supplementary Table 1. List of cytosolic proteins tested for nonAc-X²/N-degron mediated turnover in plants.

TAIR ID	UNIPROT	N-Term	Trivial name	Annotation
AT3G01290	Q9SRH6	MGNT	HIR3	SPFH/Band 7/PHB domain-containing membrane-associated protein family
AT5G17380	Q9LF46	MADKS	HACL	Thiamine pyrophosphate dependent pyruvate decarboxylase family protein
AT4G11260	Q9SUT5	MAKEL	SGT1B	Functions in plant disease resistance signaling, SCF(TIR1) mediated degradation of Aux/IAA proteins and HSP90 mediated degradation of R resistance proteins. AtSGT1a and AtSGT1b are functionally redundant in the resistance to pathogenes. AtSGT1b was more highly expressed than AtSGT1. The N-terminal TPR domain of AtSGT1a reduces the steady-state level of Arabidopsis SGT1 proteins whereas the same domain from AtSGT1b enhances SGT1 accumulation. The TPR domain is dispensable for SGT1 resistance.
AT2G47130	O80713	MSGLR	SDR3	Encodes a short-chain dehydrogenase/reductase that is not involved in ABA biosynthesis but plays an important role in plant defense response to bacteria
AT3G20050	P28769	MSISA	CCT1	Encodes a putative cytoplasmic chaperonin that is similar to mouse Tcp-1 (t complex polypeptide 1)
AT2G29720	O82384	MSLVP	CTF2B	FAD/NAD(P)-binding oxidoreductase family protein
AT5G02490	P22954	MAGKG	HSP70-2	Encodes a Heat shock protein 70 (Hsp 70) family protein;(source:Araport11)
AT1G80460	Q9M8L4	MAKEN	NHO1	Encodes a protein similar to glycerol kinase, which converts glycerol to glycerol 3-phosphate and performs a rate-limiting step in glycerol metabolism.
AT2G16600	Q38900	MATNP	CYP19	Encodes cytosolic cyclophilin ROC3. The mRNA is cell-to-cell mobile.
AT1G12780	Q42605	MGSSV	UGE1	Encodes a UDP-glucose epimerase that catalyzes the interconversion of the sugar nucleotides UDP-glucose UDP-galactose via a UDP-4-keto-hexose intermediate. Responsive to stress.

Supplementary Table 2. List of applied primers.

Primer ID	5' -> 3' Sequence	Purpose
SAT5_tFT_fwd	GACTGGTACCATGCCACCGGCCGGAGAAC	cloning of SAT5 to test its protein stability with tFT
SAT5_tFT_rev	GACTCTCGAGTATGATGTAATCTGACCATTCCGAG	cloning of SAT5 to test its protein stability with tFT
TUBB4_tFT_fwd	GATCCGTCTCGGTACCATGAGAGAGATCCTTCATATCCAAG	cloning of TUBB4 to test its protein stability with tFT
TUBB4_tFT_rev	GATCCTCGAGAGTCTCGTACTCCTCTTCTTC	cloning of TUBB4 to test its protein stability with tFT
OASTLA_tFT_fwd	GACTGGTCTCGGTACCATGGCCTCGAGAATTGCTAAAG	cloning of OASTLA to test its protein stability with tFT
OASTLA_tFT_rev	GACTGGTCTCCTCGAGAGCCTCGAAGGTCATGGCTTC	cloning of OASTLA to test its protein stability with tFT
CCT1_tFT_fwd	GACTGGTACCATGTCGATCTCCGCCCAAAATC	cloning of CCT1 to test its protein stability with tFT
CCT1_tFT_rev	GACTCTCGAGTTCTTCGCCTTGGCTCTCATC	cloning of CCT1 to test its protein stability with tFT
HIR3_tFT_fwd	GCATGGTACCATGGGGAATCTTTTCTGTTGCG	cloning of HIR3 to test its protein stability with tFT
HIR3_tFT_rev	GACTCTCGAGGGAGGCATTGTTGGCCTGTAA	cloning of HIR3 to test its protein stability with tFT
HIR3_tFT_pro_fwd	GCATGGTACCATGGGGCCAAATCTTTTCTGTTG	cloning of HIR3 with proline to test its protein stability with tFT
HACL_tFT_fwd	GATCGGTACCATGGCGGATAAATCAGAAACCAC	cloning of HACL to test its protein stability with tFT
HACL_tFT_rev	GATCCTCGAGGTTCTTGTGCTGTAATCTCCC	cloning of HACL to test its protein stability with tFT
HACL_pro_ tFT_fwd	GATCGGTACCATGGCGCCAGATAAATCAGAAAC	cloning of HACL with proline to test its protein stability with tFT
SGT1_tFT_fwd	GATCGGTACCATGGCCAAGGAATTAGCAGAG	cloning of SGT1 to test its protein stability with tFT
SGT1_tFT_rev	GATCCGTCTCCTCGAGATACTCCCACTTCTTGAGCTC	cloning of SGT1 to test its protein stability with tFT
SGT1_pro_tFT_fwd	GATCGGTACCATGGCCCCAAAGGAATTAGCAG	cloning of SGT1 with proline to test its protein stability with tFT
SDR3_tFT_fwd	GATCGGTACCATGTCGGGACTCAGATTGGATG	cloning of SDR3 to test its protein stability with tFT
SDR3_tFT_rev	GATCCTCGAGAATGGGCTTAACGACGCTATAAC	cloning of SDR3 to test its protein stability with tFT
SDR3_pro_tFT_fwd	GATCGGTACCATGTCGCCAGGACTCAGATTG	cloning of SDR3 with proline to test its protein stability with tFT
CTF2B_tFT_fwd	GACTGGTACCATGTCTCTTGTGCGTCCGATC	cloning of CTF2B to test its protein stability with tFT
CTF2B_tFT_rev	GACTCTCGAGAAGTTTGTGTGTTCCATCGTTGG	cloning of CTF2B to test its protein stability with tFT
CTF2B_pro_TFT_fwd	GACTGGTACCATGTCTCCACTTGTGCGTCCG	cloning of CTF2B with proline to test its protein stability with tFT
HSP70-2_tFT_fwd	GATCGGTACCATGGCTGGTAAAGGAGAAGGTC	cloning of HSP70-2 to test its protein stability with tFT
HSP70-2_tFT_rev	GATCCTCGAGGTCGACTTCCTCGATCTTGG	cloning of HSP70-2 to test its protein stability with tFT
HSP70-2_pro_tFT_fwd	GATCGGTACCATGGCTCCAGGTAAAGGAGAAG	cloning of HSP70-2 with proline to test its protein stability with tFT
NHO1_tFT_fwd	GCATGGTACCATGGCAAAAGAAAATGGATTTATAGG	cloning of NHO1 to test its protein stability with tFT
NHO1_tFT_rev	GACTCTCGAGGATAGAGAGGTCAGCGAGATC	cloning of NHO1 to test its protein stability with tFT
NHO1_pro_tFT_fwd	GCATGGTACCATGGCACCAAAAGAAAATGGATTTATAGG	cloning of NHO1 with proline to test its protein stability with tFT
CYP19_tFT_fwd	GACTGGTCTCGGTACCATGGCAACAAACCCTAAAGTCTAC	cloning of CYP19 to test its protein stability with tFT
CYP19_tFT_rev	GACTCTCGAGAGAAATCTGACCACAATCAGCAATG	cloning of CYP19 to test its protein stability with tFT
CYP19_pro_tFT_fwd	GACTGGTCTCGGTACCATGGCACCAACAAACCCTAAAGT	cloning of CYP19 with proline to test its protein stability with tFT
UGE1_tFT_fwd	GATCGGTACCATGGGTTCTTCTGTGGAGCAG	cloning of UGE1 to test its protein stability with tFT
UGE1_tFT_rev	GATCCTCGAGAAGCTTATTCTGGTAACCCCATG	cloning of UGE1 to test its protein stability with tFT
UGE1_pro_tFT_fwd	GATCGGTACCATGGGTCCTTCTTCTGTGGAG	cloning of UGE1 with proline to test its protein stability with tFT

Supplementary Table 2. List of applied peptides.

Peptide ID	Sequence
ASRI	ASRIARDRWGRPVGRRRRPVRVYP
APSRI	APSRIARDRWGRPVGRRRRPVRVYP
ADRS	ADRSETTRWGRPVGRRRRPVRVYP
AREL	ARELAERRWGRPVGRRRRPVRVYP
GNLF	GNLFCCVRWGRPVGRRRRPVRVYP
GPNLF	GPNLFCCVRWGRPVGRRRRPVRVYP
SGRD	SGRDGRIRWGRPVGRRRRPVRVYP
SLVR	SLVRPIYRWGRPVGRRRRPVRVYP
MPQP	MQPTETSRWGRPVGRRRRPVRVYP

References list

1. Linster E, *et al.* Downregulation of N-terminal acetylation triggers ABA-mediated drought responses in Arabidopsis. *Nat Commun* **6**, 7640 (2015).
