# Supplementary material for: Cotranslational N-degron masking by acetylation promotes proteome stability in plants
Source: Nat Commun. 2022 Feb 10;13:810. doi: 10.1038/s41467-022-28414-5 (PMC8831508; doi:10.1038/s41467-022-28414-5)
Supplement: Supplementary file 3 — Description of Additional Supplementary Files [file 41467_2022_28414_MOESM3_ESM.pdf]

Description of Additional Supplementary Files

File name: Supplementary Data 1

Description: Mass-spectrometry based identification of ubiquitinated proteins in leaves of wild type and NatA depleted plants. Wild type (WT) and NatA depleted plants (amiNAA10) were grown on soil under short day conditions. The ubiquitome of leaves from wild type and mutants were determined by LC-MS/MS after selective enrichement of proteins with the Ubi-Qapture Q matrix. The signal intensity for proteins (LFQ.intensity) is shown for individual replicates (N = 3). The first 10 amino acids (N-Terminus) of the identified proteins (TAIR ID) are shown and the proteins were classified on the basis of their N-terminal sequence as substrates of the N-acetyltransferases A-F (Nat Substrate). Proteins that are not predicted to be subject of NTA are labelled with an X. p-values were determined using the student t-test.

File name: Supplementary Data 2

Description: List of proteins (232) that were bona fide more ubiquitinated in NatA depleted plants ($p < 0.05$, or only found in all replicates of amiNAA10). Wild type (WT) and NatA depleted plants. p-values were determined using the student t-test.

File name: Supplementary Data 3

Description: Gene ontology enrichment analysis of proteins displaying bona fide higher poly-ubiquitination level in NatA depleted plants. Out of the 232 proteins that were more ubiquitinated in NatA depleted plants only 227 were associated with Gene ontologies. p-values were determined using the Fisher exact test.

File name: Supplementary Data 4

Description: Proteins quantified in the shotgun proteomics measurement of the amiNAA10, and WT Arabidopsis strain are listed. Fold change between amiNAA10 and wild type and the respective p-values from the multiple t-test are reported.

File name: Supplementary Data 5

Description: Proteins significantly downregulated in amiNAA10 compared to WT Arabidopsis with a permutation-based FDR < 0.01 are listed. Fold change between amiNAA10, and WT strains and the respective p-values from the multiple t-test are reported.

File name: Supplementary Data 6

Description: Mass-spectrometry based identification of actively translated proteins in leaves of wild type and NatA depleted plants. Wild type (WT) and NatA depleted plants (amiNAA10) were grown on soil under short day conditions. The translatome of leaves from wild type and mutants were determined by LC-MS/MS after selective enrichment of proteins with the Click-iT® Protein Analysis Detection Kit. The signal intensity for proteins (LFQ.intensity) is shown for individual replicates (N = 3). Untreated wild type served as background control (N = 2). The first 10 amino acids (N-Terminus) of the identified proteins (TAIR ID) are shown and the proteins were classified on the basis of their N-terminal sequence as substrates of the N-acetyltransferases A-F (Nat Substrate). Proteins that are not predicted to be subject of NTA are labelled with an X. p-values were determined using the student t-test.

File name: Supplementary Data 7

Description: List of proteins that were more translated in NatA depleted plants. Wild type (WT) and NatA depleted plants (amiNAA10) were grown on soil under short day conditions. The translatome of leaves from wild type and mutants were determined by LC-MS/MS after selective enrichment of azidohomoalanine containing proteins with the Click-iT® Protein Analysis Detection Kit. The signal intensity for proteins (LFQ.intensity) is shown for all replicates (N = 3) for both genotypes. The first 10 amino acids (N-Terminus) of the identified proteins (TAIR ID) are shown and the proteins were classified on the basis of their N-terminal sequence as substrates of the N-acetyltransferases A-F (Nat Substrate). Proteins that are not predicted to be subject of NTA are labelled with an X. p-values were determined using the student t-test.

File name: Supplementary Data 8

Description: List of NatA substrates displaying enhanced protein turnover in leaves of NatA depleted plants. Wild type and NatA depleted plants (amiNAA10) were gorown on soil under short day conditions. The ubiquitome and translatome of leaves from wild type and mutants were determined after selective enrichement of proteins by LC-MS/MS. The enrichment of proteins in the bona fide ubiqitome (U-Ratio) or the translatome (T-ratio) of NatA depleted plants when compared to wild type is provided as ratio amiNAA10/wildt type. Asterisks indicate proteins that were only found in amiNAA10 (N=3).
