# Supplementary material for: Cotranslational N-degron masking by acetylation promotes proteome stability in plants
Source: Nat Commun. 2022 Feb 10;13:810. doi: 10.1038/s41467-022-28414-5 (PMC8831508; doi:10.1038/s41467-022-28414-5)
Supplement: Supplementary file 12 — Reporting Summary [file 41467_2022_28414_MOESM12_ESM.pdf]

nature portfolio

Corresponding author(s): DBPR, NCOMMS-21-18461A

Last updated by author(s): Dec 17, 2021

Reporting Summary

Nature Portfolio wishes to improve the reproducibility of the work that we publish. This form provides structure for consistency and transparency in reporting. For further information on Nature Portfolio policies, see our Editorial Policies and the Editorial Policy Checklist.

Statistics

For all statistical analyses, confirm that the following items are present in the figure legend, table legend, main text, or Methods section.

n/a	Confirmed	
☐	☒	The exact sample size (*n*) for each experimental group/condition, given as a discrete number and unit of measurement
☐	☒	A statement on whether measurements were taken from distinct samples or whether the same sample was measured repeatedly
☐	☒	The statistical test(s) used AND whether they are one- or two-sided<br>*Only common tests should be described solely by name; describe more complex techniques in the Methods section.*
☐	☒	A description of all covariates tested
☐	☒	A description of any assumptions or corrections, such as tests of normality and adjustment for multiple comparisons
☐	☒	A full description of the statistical parameters including central tendency (e.g. means) or other basic estimates (e.g. regression coefficient) AND variation (e.g. standard deviation) or associated estimates of uncertainty (e.g. confidence intervals)
☐	☒	For null hypothesis testing, the test statistic (e.g. *F*, *t*, *r*) with confidence intervals, effect sizes, degrees of freedom and *P* value noted<br>*Give P values as exact values whenever suitable.*
☒	☐	For Bayesian analysis, information on the choice of priors and Markov chain Monte Carlo settings
☒	☐	For hierarchical and complex designs, identification of the appropriate level for tests and full reporting of outcomes
☒	☐	Estimates of effect sizes (e.g. Cohen's *d*, Pearson's *r*), indicating how they were calculated

Our web collection on statistics for biologists contains articles on many of the points above.

Software and code

Policy information about availability of computer code

Data collection	In this study, no custom-made software or codes/mathematical algorithms were generated by the authors. For the immunodetection of proteins, chemiluminescence signals were recorded with the ImageQuant LAS 4000 v1.2 (GE Healthcare)
Data analysis	In this study, no custom-made software or codes/mathematical algorithms were generated by the authors. For the quantification of Western Blots, the IMAGE QUANT TL v8 software package (GE Healthcare) was used. Mass spectrometry data were analyzed using the MaxQuant software, version 1.6.1.13 (https://www.maxquant.org/) and the Perseus software, version 1.6.13.0 (https://maxquant.net/perseus/). Gene Set Enrichment Analysis (GSEA) was performed by using DAVID Bioinformatics Resources version number 6.8 (https://david.ncifcrf.gov). The SIGMA Plot12 software suite was used for the statistical analysis of data. Images were processed with Fiji (http://fiji.sc). The significance of enriched NatA substrates in the different MS quantifications was calculated using the Easy Fisher Exact Test (https://www.socscistatistics.com).

For manuscripts utilizing custom algorithms or software that are central to the research but not yet described in published literature, software must be made available to editors and reviewers. We strongly encourage code deposition in a community repository (e.g. GitHub). See the Nature Portfolio guidelines for submitting code & software for further information.

Data

Policy information about availability of data

All manuscripts must include a data availability statement. This statement should provide the following information, where applicable:

- Accession codes, unique identifiers, or web links for publicly available datasets
- A description of any restrictions on data availability
- For clinical datasets or third party data, please ensure that the statement adheres to our policy

Mass-spectrometry based proteomics data determining the ubiquitome, the translatome, and the steady-state levels of leaf-proteins in the wild type and NatA depleted lines are deposited in the ProteomeXchange Consortium (http://proteomecentral.proteome exchange.org) via the PRIDE repository (https://www.ebi.ac.uk/pride/) with the data set identifiers PXD024329, PXD024328 and PXD022122, respectively

Field-specific reporting

Please select the one below that is the best fit for your research. If you are not sure, read the appropriate sections before making your selection.

☒ Life sciences ☐ Behavioural & social sciences ☐ Ecological, evolutionary & environmental sciences

For a reference copy of the document with all sections, see nature.com/documents/nr-reporting-summary-flat.pdf

Life sciences study design

All studies must disclose on these points even when the disclosure is negative.

Sample size	Sample sizes are defined in the material and method section and/or the figure caption for each experiment.
Data exclusions	No data has been excluded.
Replication	Number of replications are defined in the material and method section and/or the figure caption for each experiment.
Randomization	Plants were grown on trays in a randomized fashion.
Blinding	Blinding was not appropriate with the experimental setups used in this study.

Reporting for specific materials, systems and methods

We require information from authors about some types of materials, experimental systems and methods used in many studies. Here, indicate whether each material, system or method listed is relevant to your study. If you are not sure if a list item applies to your research, read the appropriate section before selecting a response.

Materials & experimental systems

n/a	Involved in the study	
☐	☒	Antibodies
☒	☐	Eukaryotic cell lines
☒	☐	Palaeontology and archaeology
☒	☐	Animals and other organisms
☒	☐	Human research participants
☒	☐	Clinical data
☒	☐	Dual use research of concern

Methods

n/a	Involved in the study	
☒	☐	ChIP-seq
☒	☐	Flow cytometry
☒	☐	MRI-based neuroimaging

Antibodies

Antibodies used

Custom-made α-CUL1 (anti-CUL1, 1:1000, Reference 47)
Custom-made α-O-Acetylserine(thiol)lyase A (OAS TL-A, 1:5000, Reference 48)
Custom-made α-Glutathione Reductase 1 (GR1, 1:2500, Reference 49)
Commercial α-Regulatory particle subunit 10 (RPN10, 1:2000, AS01012, Agrisera)
Commercial α-tubulin (1:5000, AS10681, Agrisera)
Commercial α-Coronatine Insensitive Protein 1 (COI, 1:1000, AS122637, Agrisera)
Custom-made α-Serine acetyltransferase 5 (SAT5,1:5000, Reference 50)
Commercial mono- and poly-ubiqitination antibody (FK2, HRP conjugate, Enzo life Sciences, dilution 1:5,000)
Commercial α-UBQ11 (1:5000, AS08307, Agrisera)
Commercial anti-S6k-p (Cell Signaling, 9205, 1:5000),

Commercial anti-S6K1/2 (1:5000, AS121855, Agrisera)

Validation

Validation of the here applied custom-made antibodies were performed in previous published studies as indicated above and in the material and method section of the manuscript. The commercial antibodies were validated by the respective company (see product-information sheets) and are routinely applied in diverse published studies.
